# A novel mutation in *CRYAB* associated with autosomal dominant congenital nuclear cataract in a Chinese family

**Published:** 2009-07-10

**Authors:** Qiang Chen, Junjie Ma, Ming Yan, Maneo Emily Mothobi, Yuanyuan Liu, Fang Zheng

**Affiliations:** 1 Center for Gene Diagnosis, Zhongnan Hospital, Wuhan University, Wuhan, China; 2 Department of Ophthalmology, Zhongnan Hospital, Wuhan University, Wuhan, China; 3 Health Research and Laboratory Services, Maseru, Lesotho

## Abstract

**Purpose:**

To identify the genetic defects associated with autosomal dominant congenital nuclear cataract in a Chinese family.

**Methods:**

Clinical data were collected, and the phenotypes of the affected members in this family were recorded by slit-lamp photography. Genomic DNA was isolated from peripheral blood. Mutations were screened in cataract-associated candidate genes through polymerase chain reaction (PCR) analyses and sequencing. Structural models of the wild-type and mutant αB-crystallin were generated and analyzed by SWISS-MODEL.

**Results:**

Mutation screening identified only one heterozygous G→A transition at nucleotide 32 in the first exon of αB-crystallin (*CRYAB*), resulting in an amino acid change from arginine to histidine at codon 11 (R11H). This mutation segregated in all available affected family members but was not observed in any of the unaffected persons of the family. The putative mutation disrupted a restriction site for the enzyme, Fnu4HI, in the affected family members. The disruption, however, was not found in any of the randomly selected ophthalmologically normal individuals or in 40 unrelated senile cataract patients. Computer-assisted prediction suggested that this mutation affected the biochemical properties as well as the structure of αB-crystallin.

**Conclusions:**

These results supported the idea that the novel R11H mutation was responsible for the autosomal dominant nuclear congenital cataract in this pedigree.

## Introduction

Hereditary congenital cataract (OMIM 604307) is an opacification of the ocular lens, which frequently results in visual impairment or even blindness during infancy or early childhood and accounts for one-tenth of the cases of childhood blindness [1,2]. Despite the great advances in the clinical management of cataracts as well as a better understanding of lens structure and function, the relationships among cataract etiology, morphology, and underlying mechanisms remain unclear. Accumulating evidence indicate that the genetic background plays an important role in the whole process. Currently, most progress has been made in identifying the genes causing autosomal dominant congenital cataract. About 39 genetic loci for congenital cataract have also been mapped, although this number is constantly increasing. Of those families with inherited cataracts for whom the mutant gene is known, about half show mutations in crystallin genes (αA-crystallin [*CRYAA*]; αB-crystallin [*CRYAB*]; βA1/A3-crystallin [*CRYBA1/A3*]; βB1-crystallin [*CRYBB1*]; βB2-crystallin [*CRYBB2*]; βB3-crystallin [*CRYBB3*]; γC-crystallin [*CRYGC*]; γD-crystallin [*CRYGD*]; γS-crystallin [*CRYGS*]), about a quarter in connexin genes (gap junction protein alpha 8 [*GJA8*]; gap junction protein alpha 3 [*GJA3*]), and the remainder in genes for heat shock transcription factor-4 (*HSF4*), major intrinsic protein (*MIP*), and beaded filament structural protein-2 (*BFSP2*) [3-7].

α-Crystallins are the most abundant soluble proteins in the lens and play essential roles in maintaining lens transparency. They are mainly composed of two related proteins, αA- and αB-crystallins, in a molar ratio of roughly 3:1. These subunits are encoded by individual genes, *CRYAA* and *CRYAB*, localized on different chromosomes and members of the small heat-shock protein family (sHSP) [8]. In the vertebrate lens, αA-and αB-crystallins form hetero-oligomers of variable size and a quaternary structure that binds and sequesters damaged proteins, preventing the formation of particulates that scatter light [9]. In contrast to αA-crystallin, αB-crystallin can also be found in tissues other than the lens and is strongly expressed in the heart, muscle, brain, and kidney. Mutations in *CRYAB* are associated with a broad variety of neurologic, cardiac, and muscular disorders, suggesting that it has a general cellular function [10-12].

In the present study, we investigated a four-generation Chinese family with autosomal dominant congenital nuclear cataract and identified a novel missense mutation in exon 1 of *CRYAB* that leads to an exchange of arginine for histidine at codon 11 (R11H).

## Methods

### Family enrollment and clinical data

**Figure 1:**
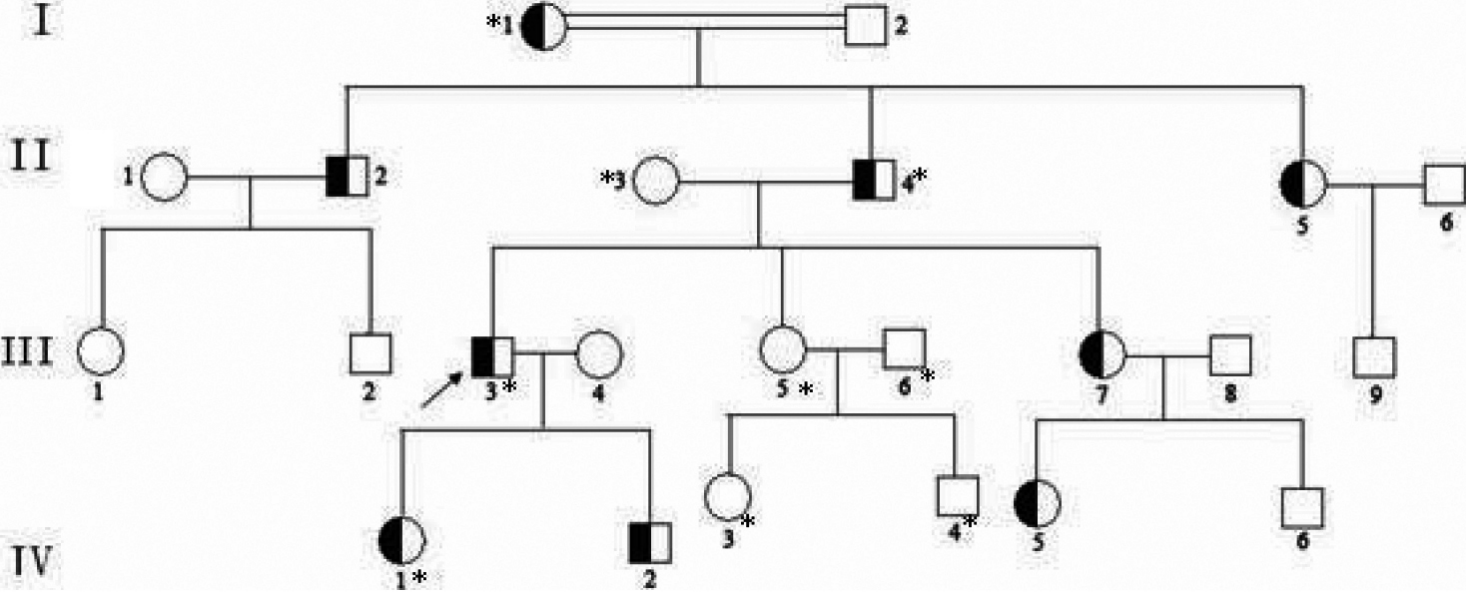
The Chinese autosomal dominant congenital cataract pedigree. Square symbols denote males, and the circle symbols denote females. The shaded symbols indicate ophthalmologist-confirmed congenital cataract. The arrow points to the proband. The transmission pattern suggested the cataract was inherited in an autosomal dominant manner. Individuals who participated in this study are indicated by an asterisk and screened for mutations.

A four-generation family (Figure 1) was recruited into Zhongnan Hospital (Wuhan, China). The research was approved by Zhongnan Hospital Research Ethics Committee and followed the tenets of the Declaration of Helsinki. Written informed consents were obtained from all the participating adult individuals and the parents of children under 16 years old. The participating subjects underwent clinical ocular examination by a senior ophthalmologist to assess the cataract phenotype through either slit-lamp photography or direct ophthalmoscope. Two hundred subjects without diagnostic features of congenital cataract and 40 subjects diagnosed with senile cataract were recruited from the Chinese Han population in Zhongnan Hospital to serve as normal controls.

### PCR based sequencing

Mutation screening was performed through candidate gene approach. Known candidate genes for hereditary cataracts such as *CRYAA*, *CRYAB*, *CRYBA1*/*A3*, *CRYBB2*, *CRYGC*, *CRYGD*, *GJA8*, *GJA3*, *MIP*, and *BFSP2* were analyzed by polymerase chain reaction (PCR) amplification followed by direct DNA sequencing. The sets of primer pairs used are listed in Table 1.

**Table 1:** Primers used for mutation screening.

**Gene symbol and amplified fragments**	**Primer sequences**	**PCR product size (bp)**	**Annealing temperature (°C)**
*CRYAA*			
Exon 1	F:5′-CTCCAGGTCCCCGTGGTA-3′	251	65
	R:5′-AGGAGAGGCCAGCACCAC-3′		
Exon 2	F:5′-CTGTCTCTGCCAACCCCAG-3′	220	65
	R:5′-CTGTCCCACCTCTCAGTGCC-3′		
Exon 3	F:5′-GGCAGCTTCTCTGGCATG-3′	309	65
	R:5′-GAGCCAGCCGAGGCAATG-3′		
*CRYAB*			
Exon 1	F:5′-TGCATATATAAGGGGCTGGCTGTA-3′	363	65
	R:3′-CAGGGTAGGAAAGGAAAATGGATG-3′		
Exon 2	F:5′-AGGATGAATTACCCGGACAGAAAG-3′	220	60
	R:5′-ACCCCTGATCCCGACTGTTAT-3′		
Exon 3	F:5′-TGAGTTCTGGGCAGGTGATAATAGTT-3′	273	60
	R:5′-AGCTTGATAATTTGGGCCTGCC-3′		
*CRYBA1/A3*	F:5′-CAATCCTCCCTCCACCTC-3′	520	57
	R:3′-TCCTTCCTTCTAGCTTTGG-3′		
*CRYBB2*			
Exon 6	F:5′-CCCCTCGTTCACCCTCCCATCA-3′	506	69
	R:5′-CACTGTGTCCAAGGTCACACAGCTAAGC-3′		
*CRYGC*			
Exons 1,2	F:5′-TCAATCATATAGACAGAGCCA-3′	784	55
	R:5′-ATCTCCATCTAACCTTAGGT-3′		
Exon 3	F:5′-AATGACAATTCCATGCCACA-3′	534	55
	R:5′-CCCACCCCATTCACTTCTTA-3′		
*CRYGD*			
Exons 1,2	F:5′-TGATAGCAATCCGAATACTCCA-3′	776	55
	R:5′-GGGTAATACTTTGCTTATGTGGGGAG-3′		
Exon 3	F:5′-GTCCTCACCAAGCTGGACTG-3′	496	55
	R:5′-CCATTTGCCTCGTGTGTGTA-3′		
*GJA8*	F:5′-AGGAGGTGAATGAGCACTCCA-3′	251	57
	R:5′-GTGCCCCACGTACATCAGG-3′		
*MIP*	F:5′-GAGGAGGTAACACTGTGGCAGC-3′	198	60
	R:3′-AGAAGCCAACGGCCAGG-3′		
*BFSP2*	F:5′-GCTGCTGCACAAACAGTTGG-3′	286	62
	R:5′-TTCTGTTTCTAATGAGGTTGAACTTGTTA-3′		
*GJA3*	F:5′-TGCAACACCCAGCAGCC-3′	474	60
	R:5′-GGCCACCGCCAGCAT-3′		

Exons of known candidate genes for hereditary cataracts were amplified from genomic DNA by PCR amplification in a 25 μl reaction volume with 10 pmol forward primers and reverse primers. F: forward primer; R: reverse primer.

The sequencing results were analyzed using Chromas (version 2.3) and compared with the reference sequence in the NCBI database. Any interesting sequence variation was later confirmed in the rest of the available family members and representative controls by restriction fragment length polymorphism (RFLP).

### PCR-RFLP analysis

**Figure 2:**
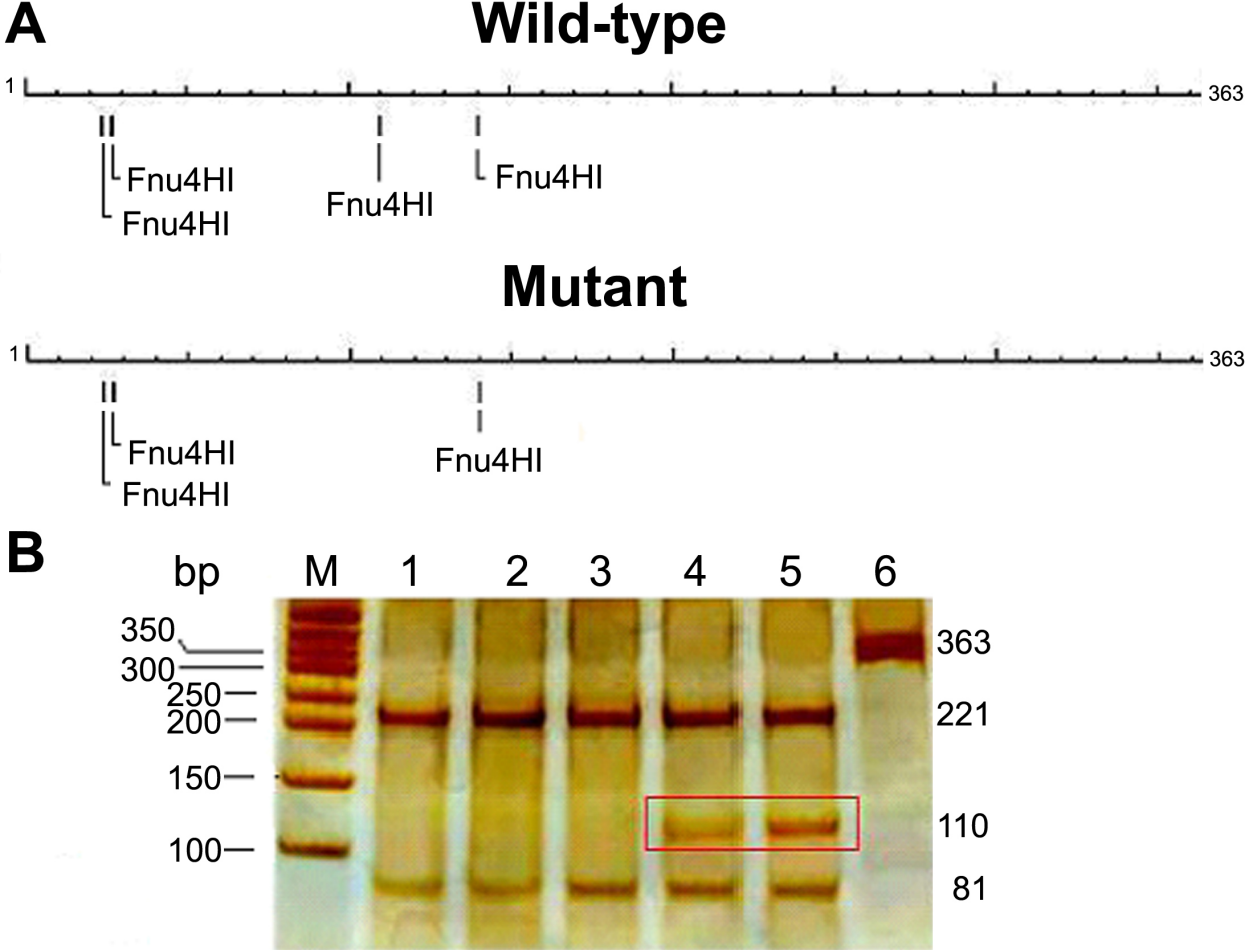
Confirmation of the mutation by PCR-RFLP method. The positions of the Fnu4HI restriction sites (GC/NGC) in the target sequence are represented (**A**). The schematic overviews show that one Fnu4HI restriction site was disrupted in the mutant form as a result of the mutation. In the wild-type form, there are two major fragments of 221 bp and 81 bp. In the disease form, one of the Fnu4HI restriction sites is disrupted, resulting in a longer fragment of 110 bp (boxed region). This longer fragment can only be observed in the affected family members (**B**). M, DNA Marker; Lane 1, unrelated normal control; Lane 2, senile cataract patient; Lane 3, unaffected member of the family; Lane 4 and 5, proband and his son; Lane 6, undigested PCR product.

RFLP analysis was used to determine whether the mutation cosegregated with the disease in the family and whether the mutation was absent in 200 randomly chosen ophthalmologically normal individuals and 40 senile cataract patients. In brief, a partial segment of *CRYAB*, which contained the putative mutation, was amplified using primers 5′-TGC ATA TAT AAG GGG CTG GCT GTA-3′ (forward primer) and 5′-CAG GGT AGG AAA GGA AAA TGG ATG-3′ (reverse primer). The PCR product was then restriction digested with Fnu4HI (MBI Fermentas, Vilnius, Lithuania) at 37 °C overnight, and the resulting fragments were separated on 8% polyacrylamide gel. The common G allele of the wild-type *CRYAB* yielded the fragments of 221 bp, 81 bp, and 30 bp while the presence of the rare A allele in the mutant form generated fragments of 221 bp, 110 bp, and 30 bp. The schema for the mutation screening by PCR-RFLP method is shown in Figure 2A.

### Molecular modeling

Biophysical predictions of the altered protein were analyzed using Bioinformatics tools. In particular, we used Antheprot 2000 (version 6.0; IBCP, Lyon, France) for secondary structures and ProtScale (provided by the Swiss Institute of Bioinformatics, Geneva, Switzerland) for hydrophilicity. Three-dimensional structures were modeled employing the Swiss Model server program (provided in collaboration by the Biozentrum; University Basel, Switzerland), the Swiss Institute of Bioinformatics (Geneva, Switzerland) and the Advanced Biomedical Computing Center (NCI Frederick, MD) [13,14] . The resulting protein database files were visualized using Swiss-Pdb Viewer (version 4.01, provided by Swiss Institute of Bioinformatics, Geneva, Switzerland) and Rasmol (version 2.7.4.2, developed by the National Science Foundation, Arlington VA). Furthermore, the resulting protein database files were calculated using Rasmol (version 2.7.4.2).

## Results

### Clinical findings

**Figure 3:**
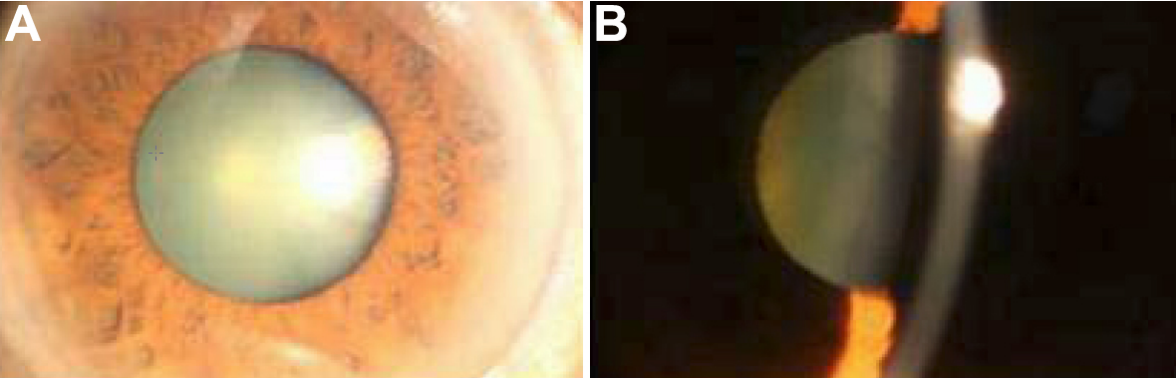
Slit-lamp photographs of the eye of the proband. Slit lamp photographs of the eye of the proband (III:3). **A**: Front view of the eye of the proband, showing cataract phenotype. **B**: Slit lamp view of the len of the proband. Lens opacities were mainly located in the nuclear area of lenses as well as in the embryonal and fetal areas.

We identified a four-generation Chinese family with 23 living members, among whom there were nine individuals affected with cataract (Figure 1). According to the history and medical records, all affected members had clinically suspected cataract before the age of five. Morphologically, all available affected individuals displayed bilateral nuclear cataracts of variable severity with no other ocular or systemic abnormalities (Figure 3). The pedigree of the family suggests an autosomal dominant mode of inheritance.

### Mutation analysis

**Figure 4:**
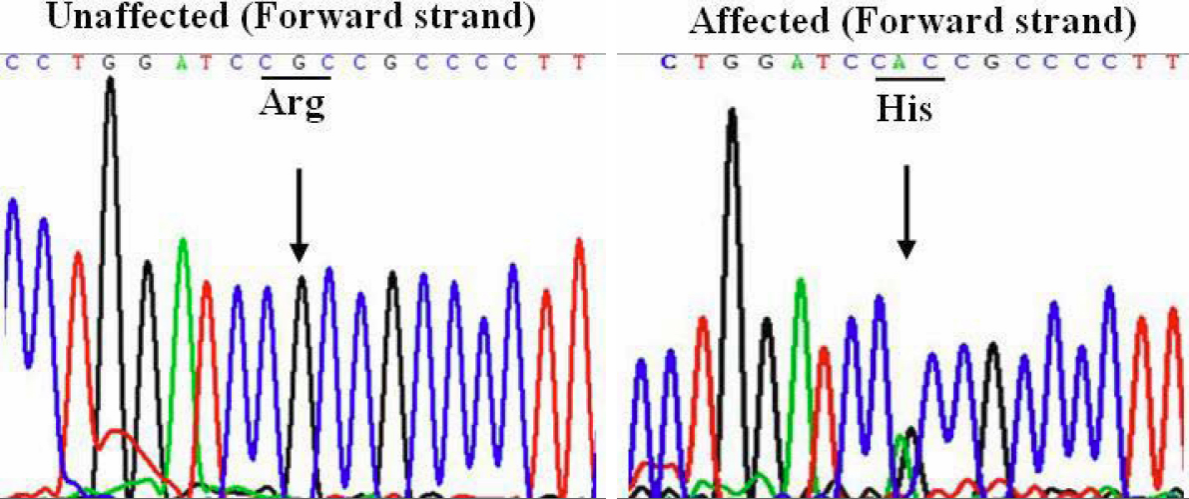
Mutation analysis of *CRYAB*. Sequence chromatograms of the partial fragment (363 bp) of *CRYAB* in one unaffected individual of the autosomal dominant congenital cataract (ADCC) family demonstrated a nucleotide sequence encoding Arg (R) at codon 11. Sequence chromatograms of one affected individual demonstrate a G to A transition resulting in an amino acid substitution of Arg by His.

To identify the mutation that caused cataract in this pedigree, we screened 16 mutation hot spots of 10 genes (Table 1) in all recruited family members by PCR-based DNA sequencing. Sequencing analysis revealed a heterozygous G→A transition at nucleotide 32 in *CRYAB*. At the protein level, it leads to an amino acid change in the first exon from arginine to histidine at codon 11 (R11H; Figure 4).

### PCR-RFLP analysis

To confirm the mutation, PCR-RFLP analysis was performed. The results showed that affected individuals carried both the wild-type allele and the mutant allele, indicating a heterozygous mutation whereas unaffected individuals in the family, unrelated normal controls, and senile cataract patients showed only the wild-type allele (Figure 2B).

### Structure predictions

**Figure 5:**
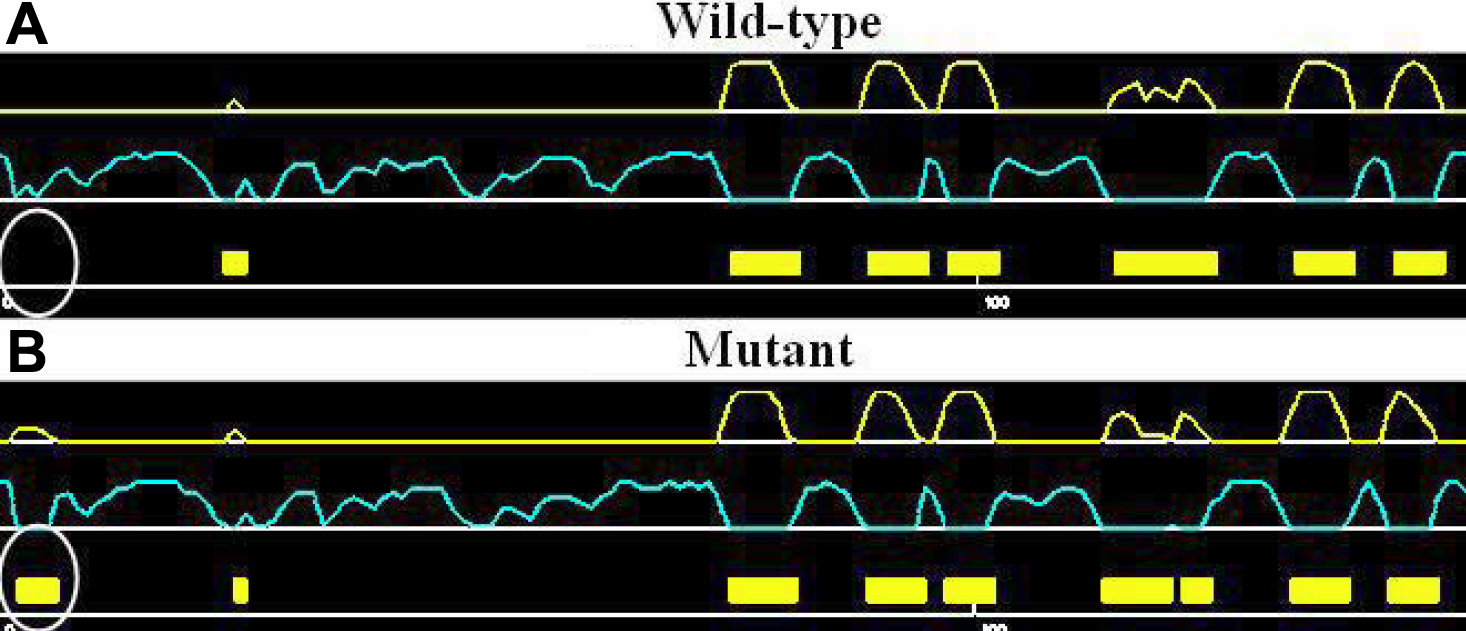
The predicted secondary structures of the wild-type and the mutant αB-crystallin. The predicted secondary structures of the wild-type form (**A**) and the mutant form (**B**) are shown. The target sequences are labeled with white circles. White: helix, Yellow: sheet, Pale blue: coil.

**Figure 6:**
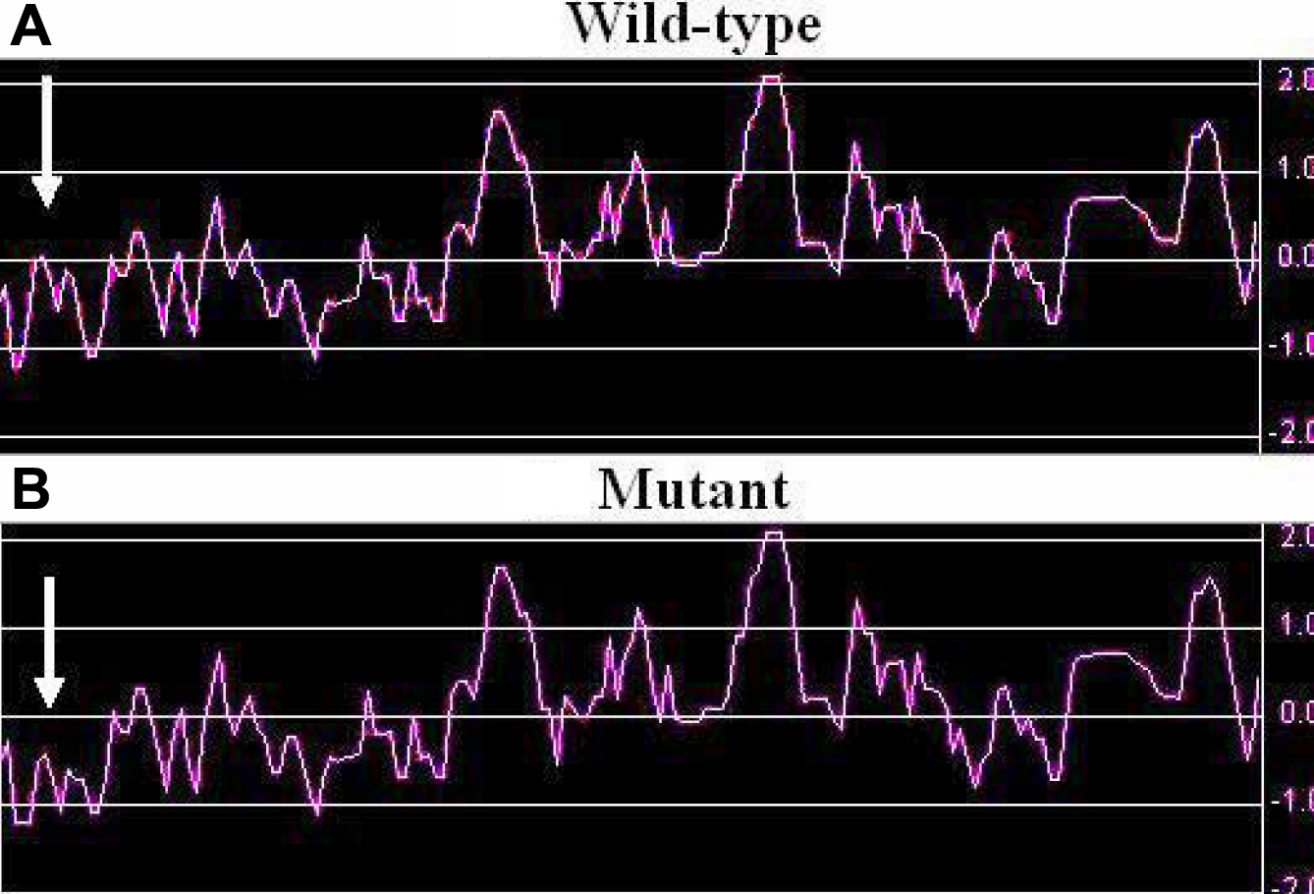
Hydropathy plot of wild-type and mutated αB-crystallin. The x-axis represents the position of amino acids. The y-axis represents the hydropathy value in a default window size of 7. It was obvious that the mutant form showed lower hydrophilicity in the corresponding region compared with the wild-type form (indicated by white arrows).

**Figure 7:**
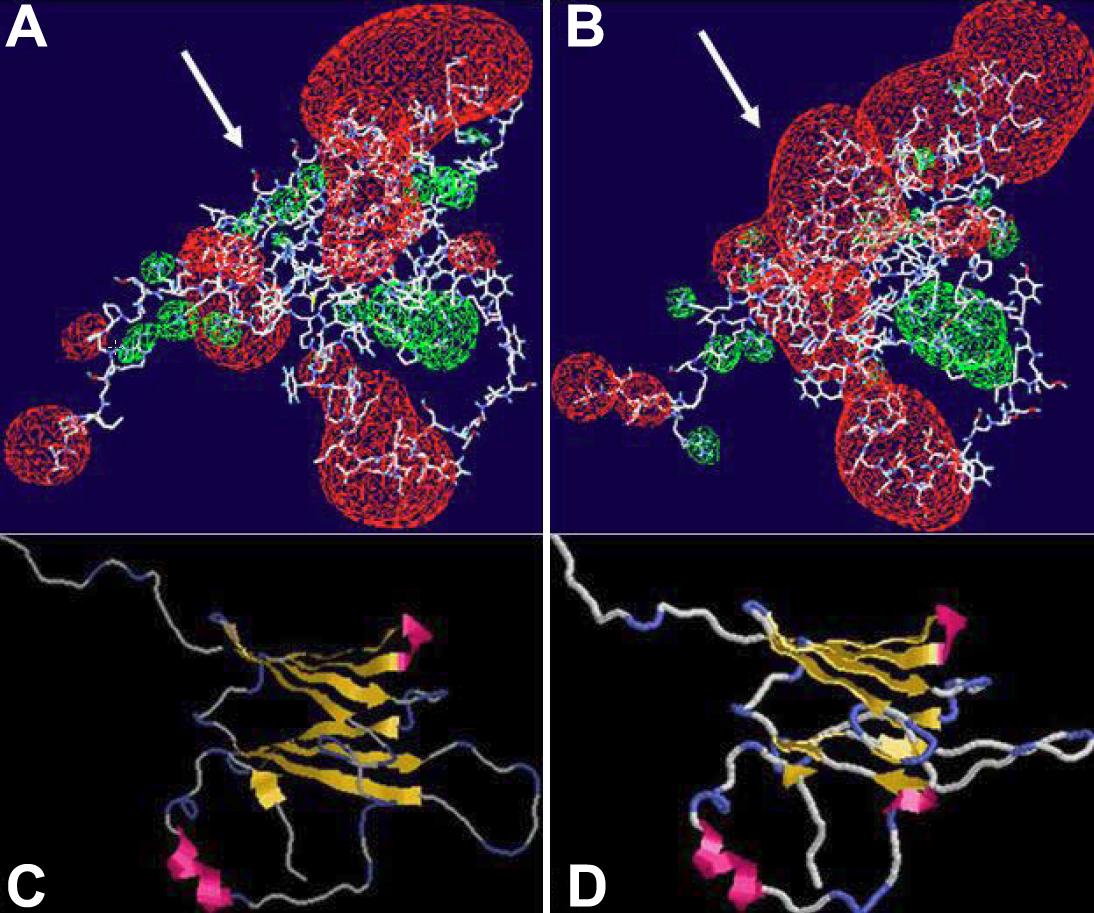
Three-dimensional protein structure. The electrostatic potentials are shown in red (negative potential) and green (positive potential) clouds. The alteration from a positive in the wild-type (**A**) to a negative potential in the mutant form (**B**) is indicated by the white arrows. Protein models of wild-type αB-crystallin (**C**) and its mutant form (**D**) are displayed. The antiparallel β sheets are yellow and the α helices are red. The blue sections are the looping regions.

Computer-assisted prediction of human αB-crystallin was performed to better understand the effects of the mutation on its biochemical properties and structure. Using the proteomics program of the Expasy Proteomics server, we compared several features between the wild-type and the mutant protein. Results by software Antheprot 2000 (version 6.0) showed that the R11H mutation caused variation of secondary structure at codon 11 (Figure 5). Moreover, the program ProtScale predicted that the hydrophilicity of the corresponding region (Figure 6) was changed as well as the isoelectric point (pI) of the entire protein (from pH 6.7 in the wild-type αB-crystallin to pH 6.5 in the mutated form). Even more striking was the alteration of the electrostatic potential (from a positive potential in wild-type to a negative potential in mutant form; Figure 7A,B) and the tertiary structure (Table 2 and Figure 7C,D).

**Table 2:** Structural characteristics of wild-type αB-crystallin and the R11H mutant form.

**αB-Crystallin species**	**H-Bonds**	**Helices**	**Strands**	**Turns**
Wild-type	76	2	8	18
Mutant	69	3	12	15

The protein database files, modeling results in Swiss Model server, were calculated by Rasmol (version 2.7.4.2).

## Discussion

In the present study, we described a novel R11H mutation in *CRYAB*, which was associated with cataractogenesis in a Chinese family. Because some members of the family failed to participate in the study, linkage analysis could not be attempted. Since this mutation segregated perfectly within this family and could not be found in representative controls, we excluded the possibility of a rare polymorphism and considered this new allele as a probable causative molecular lesion.

Lens crystallins account for nearly 90% of the total lens proteins and play essential roles in maintaining lens transparency. Therefore, mutations in the crystallin genes are strong candidates for congenital cataracts. αB-crystallin belongs to the family of small heat-shock proteins. Their characteristic features are their small size (12–43 kDa) and an α-crystallin core domain flanked by an NH_2_-terminal domain and a COOH-terminal domain [8]. The human αB-crystallin gene consists of three exons. The NH_2_-terminal domain is encoded by the first exon and the α-crystallin/heat-shock protein domain is encoded by exons 2 and 3. To our knowledge, nine mutations in *CRYAB* have been reported in the literature (listed in Table 3). Eight out of nine mutations identified in human *CRYAB* affect exon 3. Only a few of the eight mutations are associated with only dominant cataracts, and some are also suggested to be causative for desmin-related myopathy or dilated cardiomyopathy [15-22]. Based on the structure of human αB-crystallin, the R11H mutation detected in our present study lies in the NH_2_-terminal domain of αB-crystallin and resulted in dominant cataract phenotype only, which is like the P20S mutation reported previously [15]. The relationship between *CRYAB* mutations and the clinical phenotype is still unclear.

**Table 3:** Mutations in human *CRYAB*.

**Nucleotide position**	**Base-pair exchange**	**Amino acid position**	**Amino acid exchange**	**Associated pathologies**	**Reference**
32	G→A	11	Arg→His	Dominant nuclear cataract	Present study
58	C→T	20	Pro→Ser	Dominant posterior polar cataract	[15]
358	A→G	120	Arg→Gly	Desmin-related myopathy and cataract	[16]
418	G→A	140	Asp→Asn	Dominant lamellar cataract	[17]
450	delA	150	Frameshift	Dominant posterior polar cataract	[18]
451	C→T	151	Arg→stop	Desmin-related myopathy	[19]
460	G→A	154	Gly→Ser	Dilated cardiomyopathy	[20]
464	delCT	155	Frameshift	Desmin-related myopathy	[19]
470	G→A	157	Arg→His	Dilated cardiomyopathy	[21]
514	G→A	171	Ala→Thr	Dominant lamellar cataract	[22]

αB-Crystallin gene mutations identified in the present study and other previous studies which were associated with congenital cataract and/or myopathy.

It was interesting that αB-crystallin was also widely expressed in several non-ocular tissues including in the cardiac and skeletal muscle, and αB-crystallin was shown to be associated with neurologic disorders such as Alzheimer disease, Alexander disease, and amyotrophic lateral sclerosis (ALS); to participate in signaling pathways; and to protect against apoptosis [23-26]. The first *CRYAB* mutation was reported by Vicart et al. [16] who showed that desmin-related myopathy and cataract are caused by a missense mutation R120G in αB-crystallin. Animal models were generated to resolve some of the in vivo functions of α-crystallin. Brady et al [27,28]. demonstrated that targeted disruption of mouse *CRYAB* resulted in lenses similar in size to age-matched wild-type lenses with no cataracts reported. Thus, the exact in vivo molecular mechanisms by which αB-crystallin maintain lens transparency remain to be determined. More comprehensive studies will be needed to better understand the mechanism of cataract formation and the true function of αB-crystallin within the cell.

In this study, the mutant αB-crystallin predicted by the Antheprot 2000 software and Swiss Model server program showed that the R11H change not only had a significant effect on its secondary and tertiary structures but also on the hydrophilicity, isoelectric point, and electrostatic potential of the protein. As shown in Table 2, a striking consequence is the mutant form appears to have less intermolecular hydrogen-bonds, which would reduce the solubility of mutant αB-crystallin and cause cataract. The role of the NH_2_-terminal region of αB-crystallin was reported to control the species-specific assembly of subunits into higher level structures and protein interactions [29]. This is in keeping with the observation by Liu et al. [30] that the P20S mutation at the NH_2_-terminus resulted in much attenuated subunit exchange rate and chaperone activity. In addition, it was demonstrated using site-directed mutagenesis that the second residue (Asp) at the NH_2_-terminus of recombinant human αB-crystallin influenced its chaperone-like activity and hydrophobic interactions [31]. In regard to the R11H mutation in our study, we speculate based on the computer-assisted predictions that the possible mechanisms are as follows. The first possible mechanism is the substitution of Arg by His decreasing the thermodynamic stability of the NH_2_-terminal domain in a subtle way so that the protein is more prone to denaturation. Another possible mechanism is the improper folding of αB-crystallin affecting its interactions with neighboring proteins and destabilizing the complex formation critical for lens transparency. A third possible mechanism is the mutation lowering the solubility of the protein in the cytosol of the lens fiber cells and causing protein aggregation and precipitation, which would lead to cataract formation. Nevertheless, further functional experiments are necessary to explore the underlying mechanisms in details.

In summary, the present study has identified a novel missense R11H mutation in *CRYAB* that is associated with autosomal dominant congenital nuclear cataract in a four-generation Chinese family. The study further substantiates the genetic and clinical heterogeneity of congenital cataract.
